# Novel Bioreactor Design for Non-invasive Longitudinal Monitoring of Tissue-Engineered Heart Valves in 7T MRI and Ultrasound

**DOI:** 10.1007/s10439-024-03632-8

**Published:** 2024-10-22

**Authors:** Saurav Ranjan Mohapatra, Elena Rama, Maximillian P. Werner, Tobias Call, Tanja Loewenberg, Alexander Loewen, Christian Apel, Fabian Kiessling, Stefan Jockenhoevel

**Affiliations:** 1 Department of Biohybrid & Medical Textile (BioTex), Center for Biohybrid Medical Systems (CBMS), Institute for Applied Medical Engineering, RWTH Aachen University, Forckenbeckstr. 55, 52074 Aachen, Germany; 2 Institute for Experimental Molecular Imaging, RWTH Aachen University, Forckenbeckstr. 55, 52074 Aachen, Germany

**Keywords:** Tissue engineering, Bioreactor, Cardiovascular implants, Non-invasive imaging, 7T MRI

## Abstract

The development of cardiovascular implants is abundant, yet their clinical adoption remains a significant challenge in the treatment of valvular diseases. Tissue-engineered heart valves (TEHV) have emerged as a promising solution due to their remodeling capabilities, which have been extensively studied in recent years. However, ensuring reproducible production and clinical translation of TEHV requires robust longitudinal monitoring methods.

Cardiovascular magnetic resonance imaging (MRI) is a non-invasive, radiation-free technique providing detailed valvular imaging and functional assessment. To facilitate this, we designed a state-of-the-art metal-free bioreactor enabling dynamic MRI and ultrasound imaging. Our compact bioreactor, tailored to fit a 72 mm bore 7 T MRI coil, features an integrated backflow design ensuring MRI compatibility. A pneumatic drive system operates the bioreactor, minimizing potential MRI interference. The bioreactor was digitally designed and constructed using polymethyl methacrylate, utilizing only polyether ether ketone screws for secure fastening. Our biohybrid TEHV incorporates a non-degradable polyethylene terephthalate textile scaffold with fibrin matrix hydrogel and human arterial smooth muscle cells.

As a result, the bioreactor was successfully proven to be MRI compatible, with no blooming artifacts detected. The dynamic movement of the TEHVs was observed using gated MRI motion artifact compensation and ultrasound imaging techniques. In addition, the conditioning of TEHVs in the bioreactor enhanced ECM production. Immunohistology demonstrated abundant collagen, α-smooth muscle actin, and a monolayer of endothelial cells throughout the valve cusp. Our innovative methodology provides a physiologically relevant environment for TEHV conditioning and development, enabling accurate monitoring and assessment of functionality, thus accelerating clinical acceptance.

**Supplementary Information:**

The online version contains supplementary material available at 10.1007/s10439-024-03632-8.

## Introduction

The unmet clinical needs caused by cardiovascular diseases such as endocarditis and calcification have led to a substantial increase in heart valve replacements worldwide. Traditional prosthetic heart valves, though effective, have notable limitations, including thrombosis risk, structural deterioration, and lack of growth potential in younger patients. Mechanical valves, while durable, require lifelong anticoagulation, which raises the risk of bleeding. Alternatively, bioprosthetic valves are susceptible to structural deterioration, especially in younger patients, often leading to the need for reoperation [1, 2]. Consequently, cardiovascular tissue engineering has emerged as a promising solution to address these challenges, aiming to replicate the structure, function, and dynamics of native heart valves while promoting long-term integration and functionality within the body [3–6]. However, integrating tissue-engineered valves into the recipient’s body is highly complex. It poses crucial challenges, such as the degradation of synthetic scaffolds and the remodeling and maturation of the engineered tissue. For this reason, the translation of the various existing concepts into clinical applications has not yet taken place. To assess the safety and performance of a newly developed implant, it is important to monitor the entire life cycle. This starts with the in vitro conditioning phase to observe quality parameters to define release criteria and ends with clinical follow-up examinations of the implant in the patient.

In recent decades, cardiovascular MRI and ultrasound have emerged as non-invasive, radiation-free alternatives for valvular heart conditions, providing detailed valve images and precise stenosis/regurgitation assessment [7]. While non-invasive tests like ECG and auscultation remain effective, their outcomes hinge on both physician and patient [8].

With the objective of advancing biohybrid tissue-engineered implants for clinical application, our research group demonstrated the MR imaging of tissue-engineered vascular grafts both in vitro and in vivo [9]. Subsequently, we presented the imaging concept of monitoring the deposition of extracellular matrix (ECM) components and the onset of potential inflammatory reactions in vascular grafts in 7 T MRI and ultrasound imaging modalities [10, 11].

MRI offers distinct advantages over other imaging modalities like CT and X-rays for in vitro heart valve imaging. It provides excellent soft-tissue contrast and greater penetration depth, allowing for highly detailed and accurate assessments of heart valve structures. In addition, MRI does not involve ionizing radiation, which is beneficial for potential human applications. However, it is important to note that CT technology is continuously evolving, with improvements in resolution and imaging speed making it an increasingly competitive option, despite its reliance on radiation [12–14]. Recognizing the increasing demand for comprehensive valve replacement solutions, we have extended these imaging techniques to the evaluation of heart valves. However, imaging of heart valves presents distinct challenges compared to vascular grafts due to their intricate structure and dynamic movement.

Several attempts have been made to create bioreactors for conditioning heart valves [15–20], where designs were adapted primarily for the movement of the valves and providing a suitable environment for overall cell growth.

Karim et al. developed a tissue reactor for decellularizing porcine heart valves and then recellularizing them with human vascular cells, optimizing conditions to withstand cyclic pulmonary pressures [21]. Tefft et al. engineered a bioengineered heart valve and bioreactor that maintained cell viability for up to 2 weeks, focusing on sustaining pressure and flow conditions [22]. Similarly, Syedain et al. created fibrin-based heart valves and bioreactors, emphasizing their ability to endure cyclic pulmonary pressures [23]. However, none of these studies incorporated non-invasive imaging techniques like MRI. In contrast, our bioreactor integrates MRI capabilities, providing a significant advantage by enabling detailed, non-invasive monitoring of valve function and tissue growth, thus enhancing the overall evaluation and management of the bioreactor system.

Voss et al. (2022) have recently made remarkable functional bioreactors, but they were equipped with an actuator motor to open and close the valve, which does not qualify for MRI modality [24]. Even though there are previous approaches that used pneumatic drives [25, 26], they also did not have any non-invasive imaging possibility.

Non-invasive imaging like MRI offers the ability to acquire multiple types of imaging contrasts, and dynamic MRI enables real-time monitoring of physiological processes. 7 Tesla (7 T) MRI has emerged as a potential tool for cardiovascular imaging due to its ability to provide higher spatial resolution and tissue contrast than 3 T MRI [27], allowing for clear visualization of complex structures [28].

Therefore, our focus was on conducting tests using lab-sized and small animal-sized MRI devices (with a bore diameter of 70 to 75 mm). This approach allows for the exploration of small tissue-engineered heart valves, which is particularly relevant for studying young patients. The 7 T MRI offers clear advantages over lower field strengths like 3 T or 1.5 T, including superior resolution, higher signal-to-noise ratio (SNR), and enhanced soft-tissue contrast. These benefits enable much finer visualization of lesions, more precise detection of small anatomical details, and better assessment of subtle movements, which are crucial for detailed imaging studies. In the design of a small-bore bioreactor, we implemented an integrated backflow design, facilitating circulation from the ventricle to the aorta within a single cylindrical structure. The bioreactor comprises three essential chambers: the observation chamber for examining the valves, the fixation chamber for securing the valves, and the air chamber for compressing air.

**Fig. 1:**
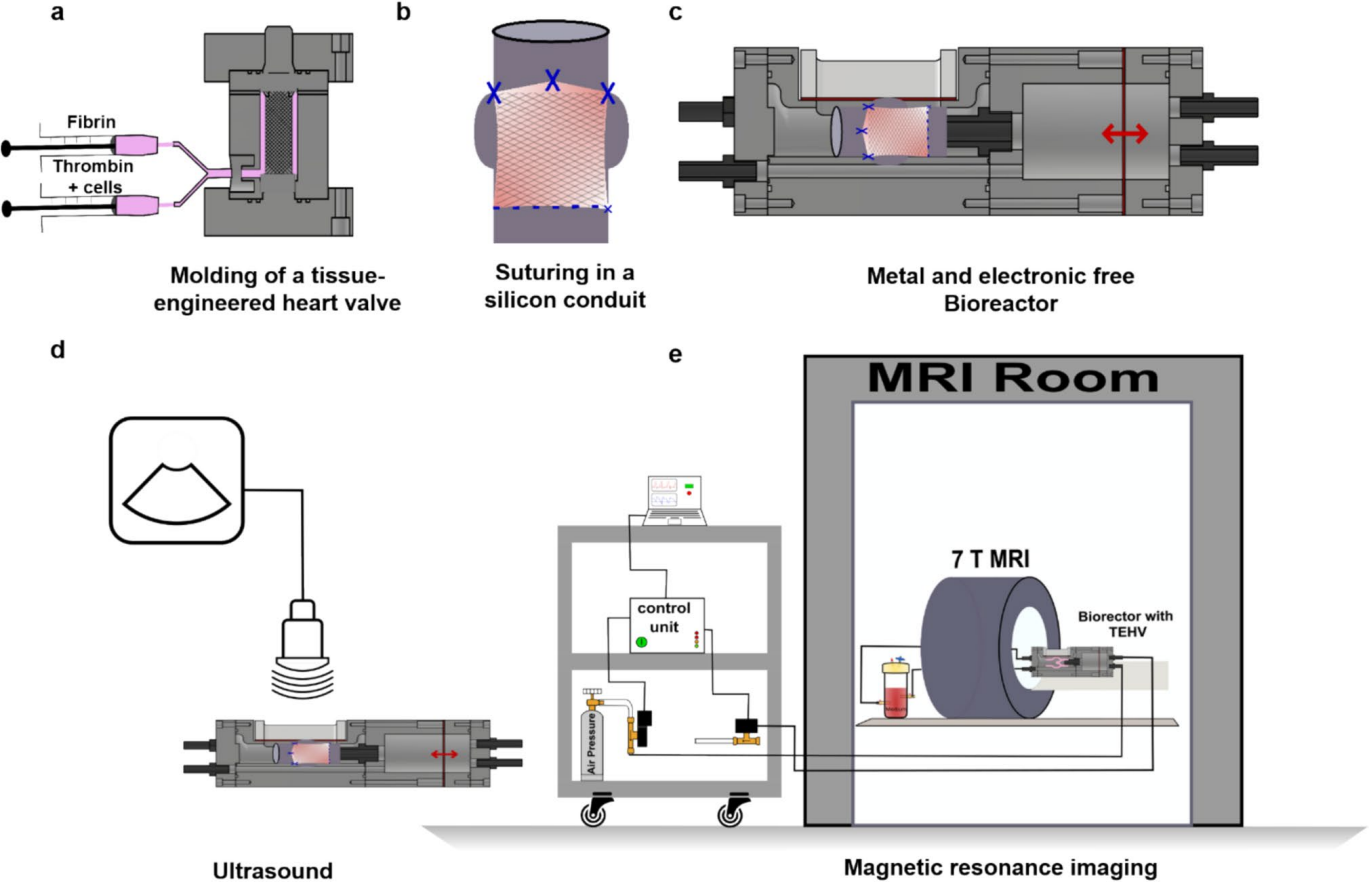
Study design. **a** Molding of a tissue-engineered heart valve with the combination of fibrin, thrombin, and arterial smooth muscle cells. **b** Tube-in-tube-like structure and suturing of the heart valve inside a silicon conduit. **c** Setup of the metal and electronic free bioreactor. **d** Ultrasound setup for the bioreactor. **e** Bioreactor and its elongated setup outside the MRI room

The TEHVs were fabricated by combining medical-grade polyethylene terephthalate (PET) textile and fibrin matrix gel along with human arterial smooth muscle cells and were sutured on a silicon conduit (Fig 1a–b) as previously described [24, 29]. To improve the detectability of the TEHVs in MRI, the PET scaffold was coated with superparamagnetic iron oxide nanoparticles (SPION)-labeled polylactic glycol acid (PLGA) electrospun fibers.

In this study, we present a novel bioreactor design for the conditioning of aortic tissue-engineered heart valves (TEHV) that supports multi-modal imaging (Fig 1 c–e). We demonstrate the MRI compatibility of our bioreactor without encountering blooming artifacts, allowing for clear observation of the dynamic movement of TEHVs using gated MRI motion artifact compensation and ultrasound imaging techniques. Furthermore, our bioreactor’s role in enhancing extracellular matrix (ECM) production within the TEHVs by mimicking the physiological conditions of the human body by precisely controlling factors like fluid flow, temperature, CO_2_ concentration, and pressure within the bioreactor was confirmed through immunohistological analysis, revealing abundant collagen, α-smooth muscle actin (α-SMA), and endothelial cell monolayers across the valve cusp.

Where ultrasound offers real-time insights into structural aspects and immediate changes, and MRI complements the analysis by providing detailed anatomical and functional information over longer timeframes, the synergy between these two imaging modalities enhances the overall understanding of tissue development, allowing researchers to make informed decisions during the design and optimization of tissue engineering strategies. In addition, this setup can also be transferred to translational implant development and enable an entire implant life-cycle management to ensure patient safety. Looking ahead, this bioreactor serves as a ‘Blackbox’ capable of gathering and interpreting critical information such as ECM remodeling and material degradability, previously unknown, thus advancing our understanding of implant performance and longevity.

## Materials and Methods

### Production of Textile Mesh

The tubular textile mesh was produced from medical-grade polyethylene terephthalate (PET) multifilament yarn on a DR 16 EEC/EAC double-face Raschel warp knitting machine (Karl Mayer GmbH). The yarn had a yarn count of 78 dtex and consisted of 24 filaments. The yarn type was Fully Drawn Yarn (FDY) with 100% PET composition, and the breaking load of the yarn was 334 g, and the breaking elongation was 30%. A tull-filet pattern was employed while manufacturing, along with a needle gauge of E30 (equivalent to 30 needles per inch) and a course density of 15 stitches per centimeter. To create the tubular structure, 52 PET yarns were processed, and this structure was thermostabilized at 200 °C for 8 min on a metal rod before it was ready to be molded.

### Electrospinning of SPION-Labeled PLGA Fibers

Polylactic-co-glycolic acid (PLGA) (Purasorb PLG 8523, Corbion Purac Gorinchem, Netherlands) was dissolved in chloroform and methanol. The SPION particles were synthesized as described in Ref. [30] and were then mixed for 24 h with the PLGA solution at room temperature. The combination of both PLGA and SPION solution was loaded into a coaxial electrospinning needle (Bioinicia SL, Paterna, Spain) and was electrospuned for 10 min in a custom-made device. Electrospinning was performed in a constant climate chamber (Binder GmbH, KBF 720) at 25 °C and less than 30% humidity. The motor for the rotation was applied to the platform for horizontal movement. The mandrel with the PET textile was attached to it via a PEEK (polyether ether ketone) connector. A plastic holder on the open side stabilizes the mandrel. The aluminum mandrel (13- and 15-mm diameter) and PEEK connector were custom-made. For each sample, a 15-cm-long PET mesh was set on the collector rod for spinning. The stand-off distance between the collector and nozzle tip was set to 20 cm, and the motor speed was 30 mm/s. The flow rates at the core were 0.5 ml/h and at the shell 1 ml/h during the spinning process. At the nozzle, + 22 kV (emitter) and the collector, − 20 kV, were applied during the spinning.

### Scanning Electron Microscopy

For a comprehensive qualitative analysis of the fiber orientation, scanning electron microscopy (Thermo Fisher Scientific Inc., Quattro S) images were taken. The samples were coated (Leica Camera AG, High Vacuum Sputter Coater, EM SCD 500) either by gold palladium or graphite to prevent the charging of the surface. The operating voltage was set to 10 kV. Images were taken at 100-, 500-, and 1000-fold magnification. A gold–palladium coating was employed to enhance the signal-to-noise ratio during secondary electron detection mode (SE). To mitigate charging effects and guarantee electrical conductivity, a thin layer of Au/Pt was applied to the specimens before conducting SEM image measurements. SE mode images were captured with an electron acceleration voltage of 10 kV, utilizing an Everhart–Thornley detector (ETD) in a high vacuum setting at a distance of 15 cm. The precautionary step of coating the specimens with a thin layer of Au/Pt was taken to prevent charging effects and ensure optimal electrical conductivity during the SEM image measurement process.

### Cell Isolation

Smooth muscle cells and endothelial cells were isolated from human umbilical cords under the approval of the ethics committee of the human subjects (vote of the local ethics committee: 'EK 2067). A transport buffer solution was prepared to transport the human umbilical cord. The umbilical cord was kept at 4 °C for 4 h before the isolation in the transport buffer. To remove the remaining blood clots, the umbilical cord was thoroughly cleaned with PBS via an irrigation cannula. The two arteries from the umbilical cord were harvested, and all the unwanted tissue was removed carefully with tweezers. As described before [11, 31, 32], for the collection of smooth muscle cells, the arteries were extracted from the cord and finely chopped into small ring-shaped sections using a scalpel. These small segments were then evenly distributed horizontally within a T75 cell culture flask. Once the arterial rings were slightly dry and stable on the surface, they were fed with fresh DMEM medium (Thermo Fisher, Germany) containing antibiotics. Since the flask was not coated with gelatin, it was unsuitable for endothelial cells, which were washed away during the medium change the following day. Small colonies of SMCs were observed in 2 to 3 days, and 70–80% confluency was achieved in 7 to 10 days.

To isolate endothelial cells, the lumen of the HU artery was filled with collagenase (Thermo Fischer, Germany) by a cannula and incubated at 37 °C for the collagenase enzymatic reaction to happen for 30 min. Following the removal of collagenase, the cells were cultured in a flask and supplied with an endothelial basal medium (Promocell, Germany). The flasks were coated with gelatin, which was responsible for cell attachment to the surface. Cell colonies were confirmed the following day, and confluency was achieved in a week.

### Design and Production of the Bioreactor

**Fig. 2:**
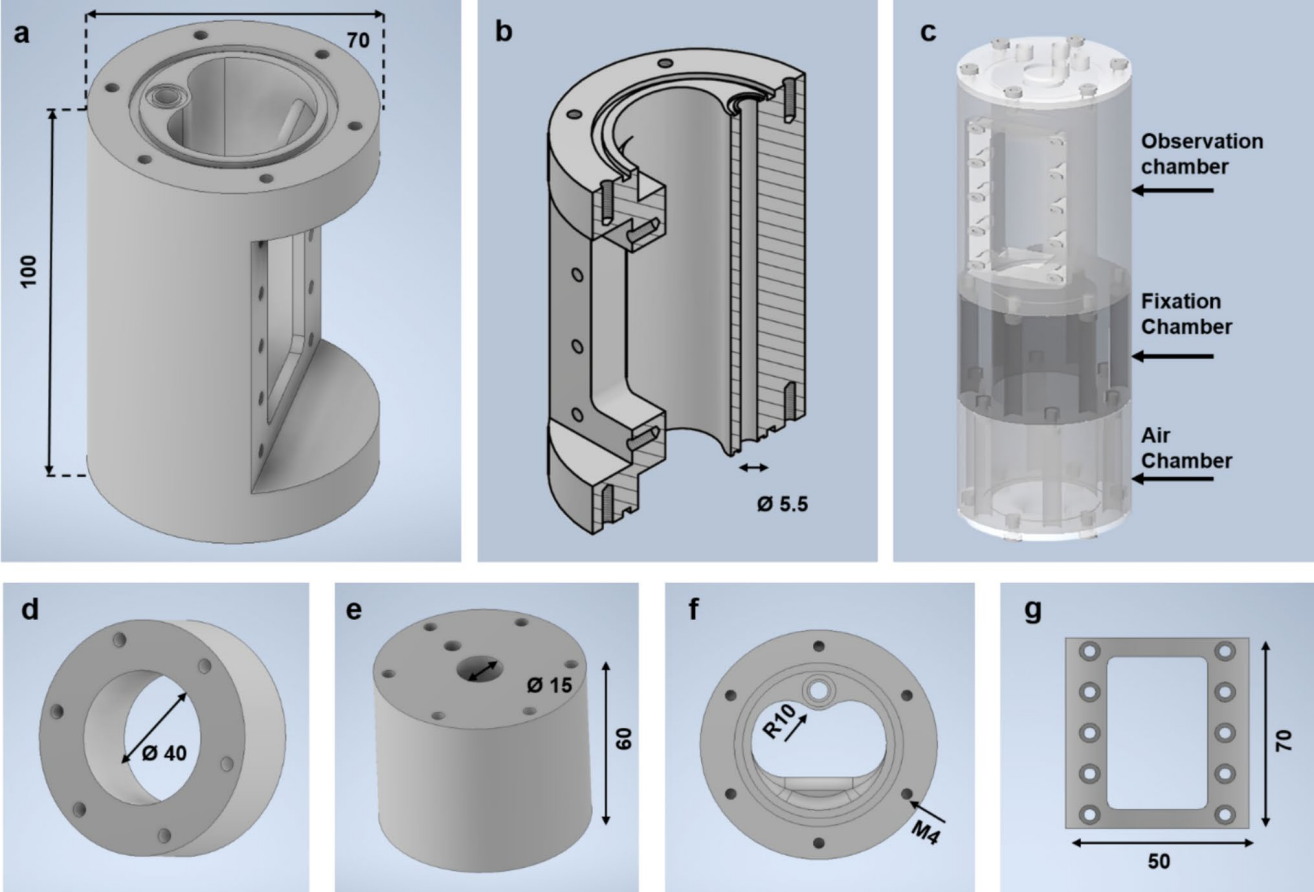
Computer-aided design of the bioreactor chambers. **a** The observation chamber with an integrated backflow. **b** Side view of the observation chamber showing the vertical cross-section. **c** Assembly of all chambers of the bioreactor. **d** Side view of air chamber. **e** Side view of fixation chamber. **f** Top view of the observation chamber. **g** The ultrasound window cap. All the dimensions are in mm

The bioreactor was designed using computer-aided design (CAD) software (Autodesk, California, USA) and produced from polymethyl methacrylate (PMMA) by CNC machine. The bioreactor was divided into three distinct chambers, each designed for a specific function (Fig 2c). The first chamber, known as the fixation chamber, was where the TEHV was fastened. This chamber operated analogously to the ventricle of the heart, simulating the conditions that the valve would experience in vivo.

The second chamber, referred to as the observation chamber, functions similarly to the aorta of the heart. It includes an ultrasound window, which allows for real-time monitoring of the valve. In addition, MRI images were captured from this chamber, providing detailed insights into the valve’s performance, hence its designation as the observation chamber.

The third chamber, the air chamber, contained only compressed air and no other medium. The pressurized air in this chamber exerts a force on a membrane, which in turn propels the medium from the fixation chamber through the TEHV, effectively simulating the flow of the medium through a heart valve. All chamber’s outer diameter was set to 70 mm as the coil in the MRI system had a 72 mm diameter. The inner diameter of each chamber was designed according to their different application, as mentioned in Fig 2a, d, and e.

The transparent nature of PMMA makes it suitable for qualitative observations of the conditioning. All parts of the bioreactor except the screws and membrane were sterilized by low-temperature hydrogen peroxide gas plasma (ASP Global Manufacturing GmbH, STERRAD 100S Sterilization System). To assemble all the chambers of the bioreactor, 20 M4x20 and 10 M4x8 polyether ether ketone (PEEK) and 6 M4x50 polyamide PA screws were used. Pieces of silicone membrane (thickness 0.44 mm) (Wacker Chemie AG) were cut to fit between the air and fixation chamber and between the observation chamber and observation window (Fig. 2g).

To avoid leakage, sealing rings were inserted at the contact areas of the remaining bioreactor parts. Three and two nozzles (Sang-A, straight hose nozzle 8 mm R 1/8" male thread PA) were added on the lid and the floor, respectively. The threads of the nozzles were covered by Teflon film to prevent leakage.

### Tissue-Engineered Heart Valve

Two cylindrical structures were engaged to make the TEHV. One is a silicon conduit that acts as a housing of the valve, and another is a tubular scaffold, which is the leaflets. This structure is based on a tube-in-tube concept, where the leaflets were stitched into a silicone housing conduit (Fig 1b). The valve was molded in a 3D-printed veroclear mold where the hydrogel and cells were injected (Fig 1a, S1). The hydrogel matrix was prepared by combining 50% fibrinogen (10 mg/mL), 7.5% thrombin (40 U/mL), 7.5% 50 mM CaCl2 in Tris buffer solution (TBS), and human umbilical artery smooth muscle cells. The cell density was 10 × 10^6^ cells/mL in a total 2 mL volume of fibrin gel for every valve. After the injection molding and the fibrin gel were allowed to polymerize completely, the TEHVs (*n* = 3) were statically conditioned for 7 days, following the procedures outlined by Moreira and Weber [29]. During this static phase, the culture medium is continuously cycled through a reservoir with gas exchange filters, ensuring a stable environment for the cells, although the valve is not yet exposed to dynamic flow conditions. This static conditioning phase is crucial for the initial growth and maturation of the ECM, making the valve more resilient and better prepared to withstand the stresses of dynamic flow introduced later in the process.

After the static culture, the valves were endothelialized inside a 50 mL falcon tube with an endothelial cell density of 3 × 10^6^/mL. To culture, both endothelial cells and smooth muscle cells, a combination of both mediums was used along with supplements. The bioreactor medium consisted of 500 mL of DMEM along with 50 mL fetal bovine serum, 5 mL antibiotics, 308 mg Vitamin C, and 1712 μL tranexamic acid, and 500 mL of Lonza EGM (Lonza Group AG, EGM-2 Microvascular Endothelial Cell Growth Medium-2 BulletKit, CC-320) with supplements and 308 mg of Vitamin C. The bioreactor runs for 7 days and is driven by a pressurized air system. A magnetic controllable valve (ODE-Magnetventile/SFS-Fluid Systeme GmbH, magnetic valve, 21A2ZV45D) was connected to a pressured air supply that provides a pressure of 1 bar and to the nozzle on the floor of the bioreactor. The second outlet of the air chamber is connected to a second controllable valve (ASCO Power Technologies, microfluidic valve, PVG202A203V.24/DC), which has an open end on the other side. Both valves were connected to a DAQ box to control the valves via the LabView program.

The bioreactor is positioned within an incubator maintained at 37 °C and a CO_2_ concentration of 5% (CB 210, BINDER GmbH, Germany). The bioreactor culture medium was manually changed every 3 days.

### Magnetic Resonance Imaging

The bioreactor setup was the only component placed inside the MRI room (Figs. 1e, 5a), with all electronic equipment and controls kept outside to avoid interference with the MRI. Compressed air required for the valve’s operation was delivered through specific air-pressure-carrying tubes, which connected the external air supply to the bioreactor inside the MRI room. This arrangement ensured that the bioreactor could function effectively within the MRI environment without compromising the imaging quality or the integrity of the electronic systems.

TEHVs were monitored using a Bruker BioSpec 70/20 USR 7 T MRI scanner (Bruker BioSpin GmbH, Germany). To reduce movement artifacts, a breathing patch was secured on top of the ultrasound membrane to synchronize MR data acquisition to the mechanically induced cardiac cycles. First, the positioning and the MRI visibility of the valve were assessed by T2-weighted sequences, which were acquired axially and coronally using a fast spin echo sequence [repetition times (TR): 209.4 ms; echo time (TE): 2.8 ms; flip angle: 50°; averages: 4; matrix size; 360 x 360; field of view (FOV): (50 x 50) mm^2^; slice thickness; 1 mm]. Subsequently, gradient-recalled echo pulse or cine sequences of the heart valves, consisting of multiple static images rapidly displayed in a continuous loop, were acquired [TR: 11.02 ms; TE: 3.9 ms; flip angle: 10°; repetitions: 4; oversampling: 100; movie frames: 15; matrix size; 256 x 256; FOV: (50 x 50) mm^2^; slice thickness; 1 mm]. Imalytics Preclinical Software (Gremse-IT GmbH, Aachen, Germany) was employed for image analyses. The maximum opening area of the valves was calculated using the Image J (NIH, USA) software.

### Ultrasound

Ultrasound was performed using a VEVO 3100 ultrasound system equipped with a linear array-MX-250 transducer (FUJIFILM VisualSonics, Toronto, Ontario, Canada). The transducer was placed vertically onto the bioreactor window positioned on top of the heart valves. The small cavity of the US window was filled with water to decrease the acoustic impedance. The heart valves were imaged in bright field mode at 21 MHz frequency and 100 frames. VevoLAB software version 5.2 (FUJIFILM VisualSonics, Toronto, Ontario, Canada) was used for image analyses.

### Immunohistochemistry

The Carnoy’s solution was used to fixate the samples based on the previously published protocol described by Koch et al. [33], which is a mixture of ethanol, chloroform, and acetic acid. Afterward, the samples were washed in ethanol for 30 min, followed by dehydration using a dehydration device (Leica TP 1020, Wetzlar, Germany). The specimens were subsequently embedded in paraffin for storage until further use. Sections of paraffin, with a thickness of 5 µm, were cut using a microtome (PFM medical, Germany). Preceding the staining process, the samples underwent deparaffinization through a sequential dilution series of xylol and ethanol. The Sequenza Staining racks (Thermofisher Scientific, Massachusetts, USA) were employed to secure the samples in position. To block and permeabilize the tissues, a 0.1% Triton X-100 aqueous solution containing 5% normal goat serum (NGS) from (Agilent Dako, Santa Clara, California, USA) was utilized. Subsequently, the primary antibodies were subjected to an incubation period at 37 °C for 1 h, followed by a triple wash with PBS for 5 min each. The samples were incubated with the corresponding secondary antibodies for 1 h. Following another round of triple washing, the samples were incubated with DAPI (1:500) from (Thermofisher Scientific, Massachusetts, USA), washed three times with PBS, and finally mounted using a fluorescent mounting medium from (Agilent Dako, Santa Clara, California, USA). The primary antibodies utilized for the histological analyses were directed against anti-human CD31 (Sigma-Aldrich, Darmstadt, Germany), anti-human alpha-smooth muscle actin (α-SMA) (Sigma-Aldrich, Darmstadt, Germany), and anti-human type I collagen (Acris Antibodies GmbH, Herford, Germany). The secondary antibodies, namely anti-mouse Alexa Fluor 488 and anti-rabbit Alexa Fluor 594, were sourced from Thermofisher Scientific (Massachusetts, USA). The immunohistology images were acquired using an Axio Imager M2 fluorescence microscope (Carl-Zeiss, Oberkochen), with a magnification of 5x for the overview of the cusp and 20x for the detailed image of the cusp.

## Results

### Electrospinning and Molding of the TEHVs

**Fig. 3:**
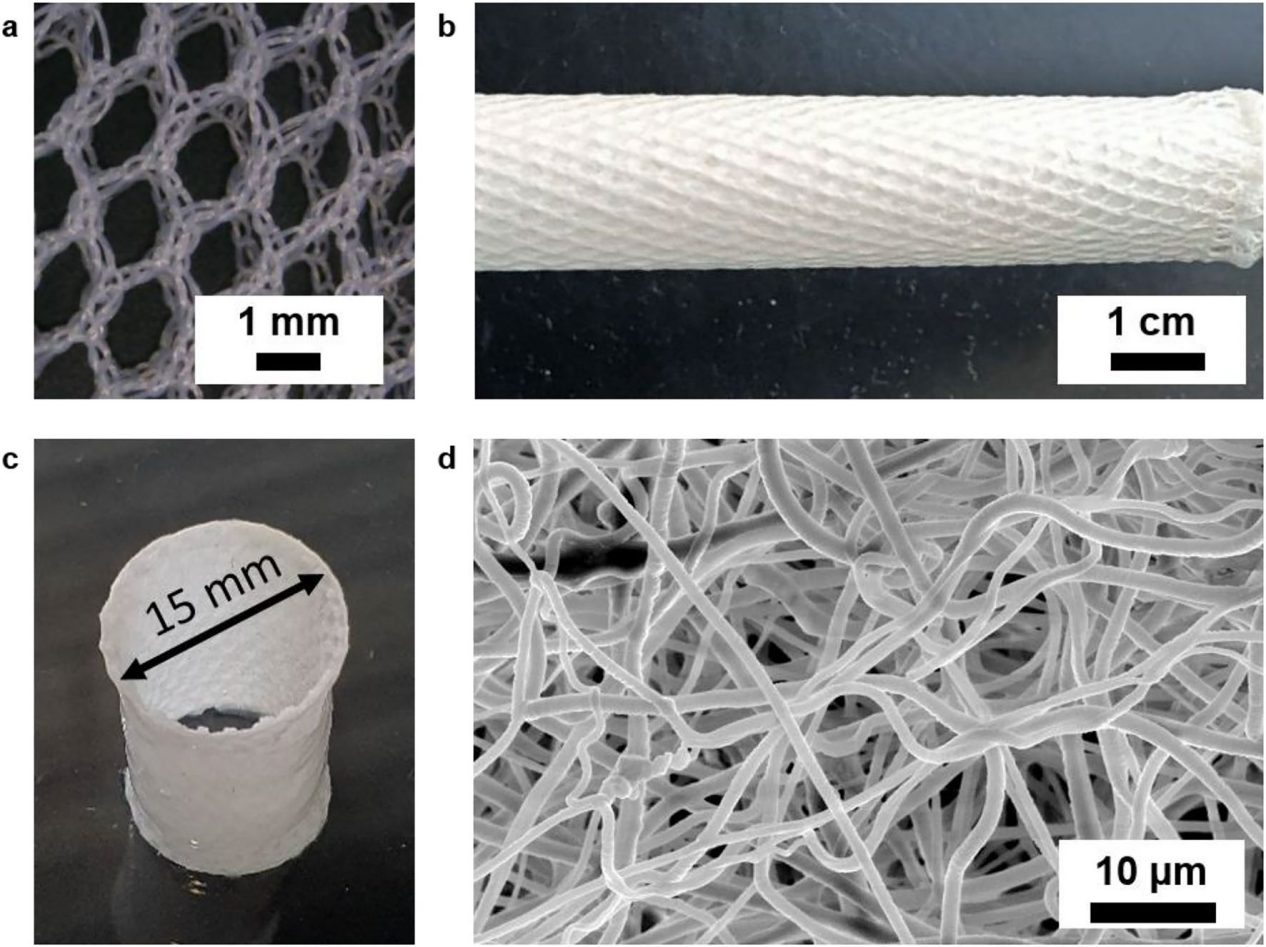
Textile mesh and the electrospun coating. **a** Wrap-knitted PET textile mesh showing the multifilament threads and tull-filet pattern. **b** The textile scaffold after electrospun coating by SPION-labeled PLGA solution. **c** A molded tube of fibrin, thrombin, and cells before it is placed and sutured inside the silicon conduit. **d** Scanning electron microscopy of the electrospun fibers representing the fiber distribution

The PET textile scaffold and the non-woven electrospun fibers from the SPION-labeled PLGA were visible under a light microscope (Fig 3a, b). However, to observe the orientation and deposition of the fibers, scanning electron microscopy (SEM) was performed. The SEM images (Fig 3d) confirmed the uniform fiber distribution and the porosity achieved with the electrospun coating. No evidence of fiber agglomeration was found. The porosity of the PET scaffold was calculated by area measurement in a microscope (Keynce, Offenbach, Germany). The porosity was 49.5% in the textile scaffold (*n* = 3, SD = 0.47).

### Bioreactor Design and Setup

**Fig. 4:**
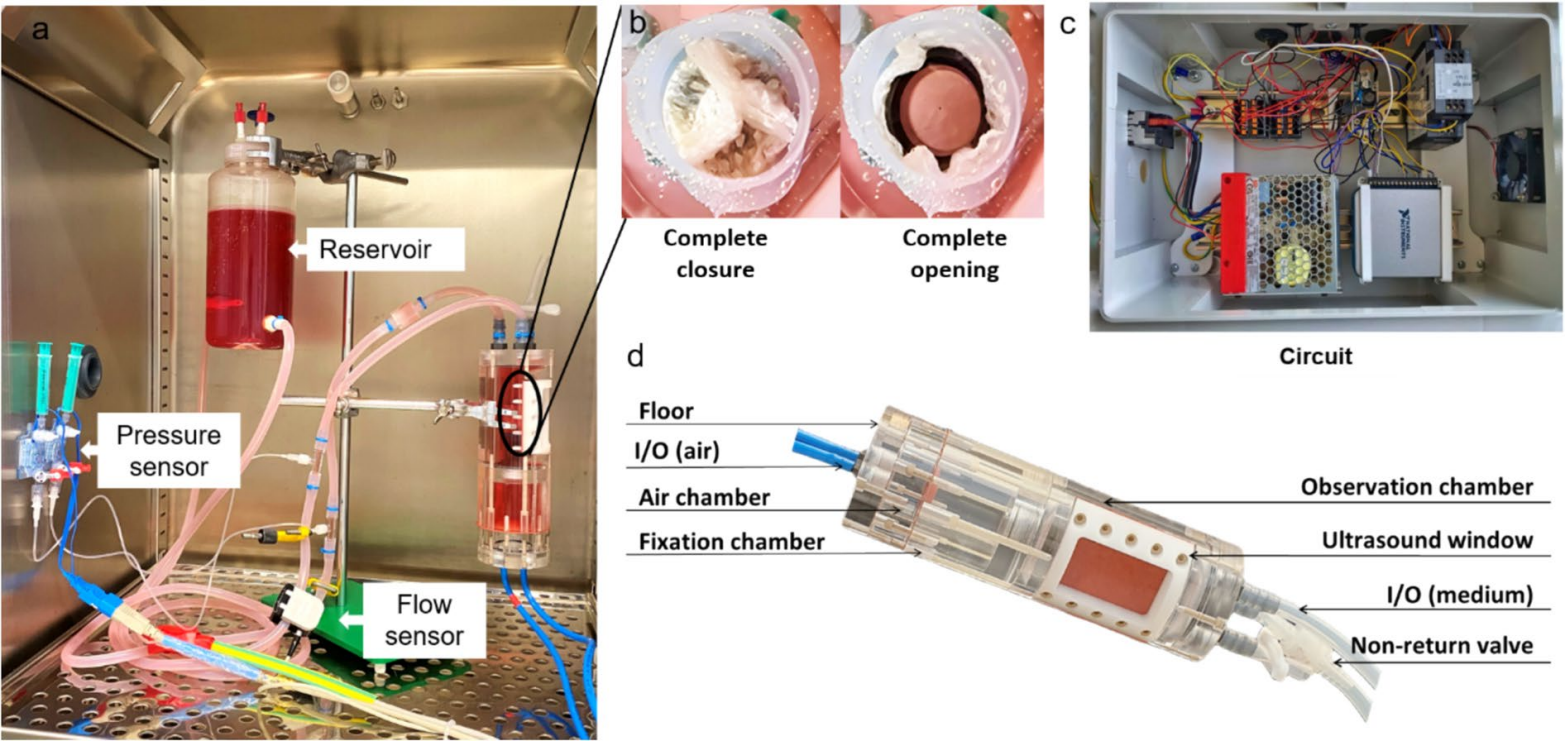
Bioreactor setup with circuits. a The bioreactor setup inside the incubator shows the components like the pressure sensor, flow sensor, and pneumatic drive channels. **b** Representative pictures of the complete opening and closing of the valves when observed from the top. **c** The circuit and data acquisition (DAQ) device that runs the bioreactor. **d** The assembly of the bioreactor shows the chambers and their fittings

The limited space imposed by the 72-mm diameter MRI coil prevented the inclusion of peripheral tubing, which is essential for ensuring a connection between the ventricular and atrial block. Consequently, an integrated backflow system was successfully designed to circumvent this need for peripheral connection (Fig 2a–c). As shown in Fig 4d, the whole bioreactor appears as a single cylinder because the connection between the ventricular and aorta block was fully integrated, which successfully resulted in placing the bioreactor in the MRI coil. The bioreactor was designed to have an outer diameter of 70 mm to fit in the MRI coil and have 1 mm on each side for free movement.

The cardiac cycle was represented in two phases: during the diastolic phase, as the membrane moved downward, the cell culture medium flowed from the ‘atrial’ reservoir into the ‘ventricular’ chamber of the bioreactor, passing through a non-return valve that mimicked the mitral valve function. In the subsequent systolic phase, the membrane was pneumatically pushed upward, displacing the medium through the ‘aortic’ valve into the ‘aorta’, and from there, it returned to the atrial reservoir, which also acted as a windkessel, simulating aortic compliance.

The closed-loop bioreactor system (Fig 4a) did not show any leakage. The mechanical valves of the pressurized air system were reliable. The tubing system between the bioreactor and medium reservoir tended to collect air, which was then removed manually afterward. The complete opening of the TEHV was achieved by considering three important factors: (1) the amount of pressure supplied to the air chamber, (2) geometry, and (3) the suturing positions of the valve. In our in vitro experiments, valves opened fully at a ventricular pressure of 20-40 mmHg.

### Magnetic Resonance Imaging

**Fig. 5:**
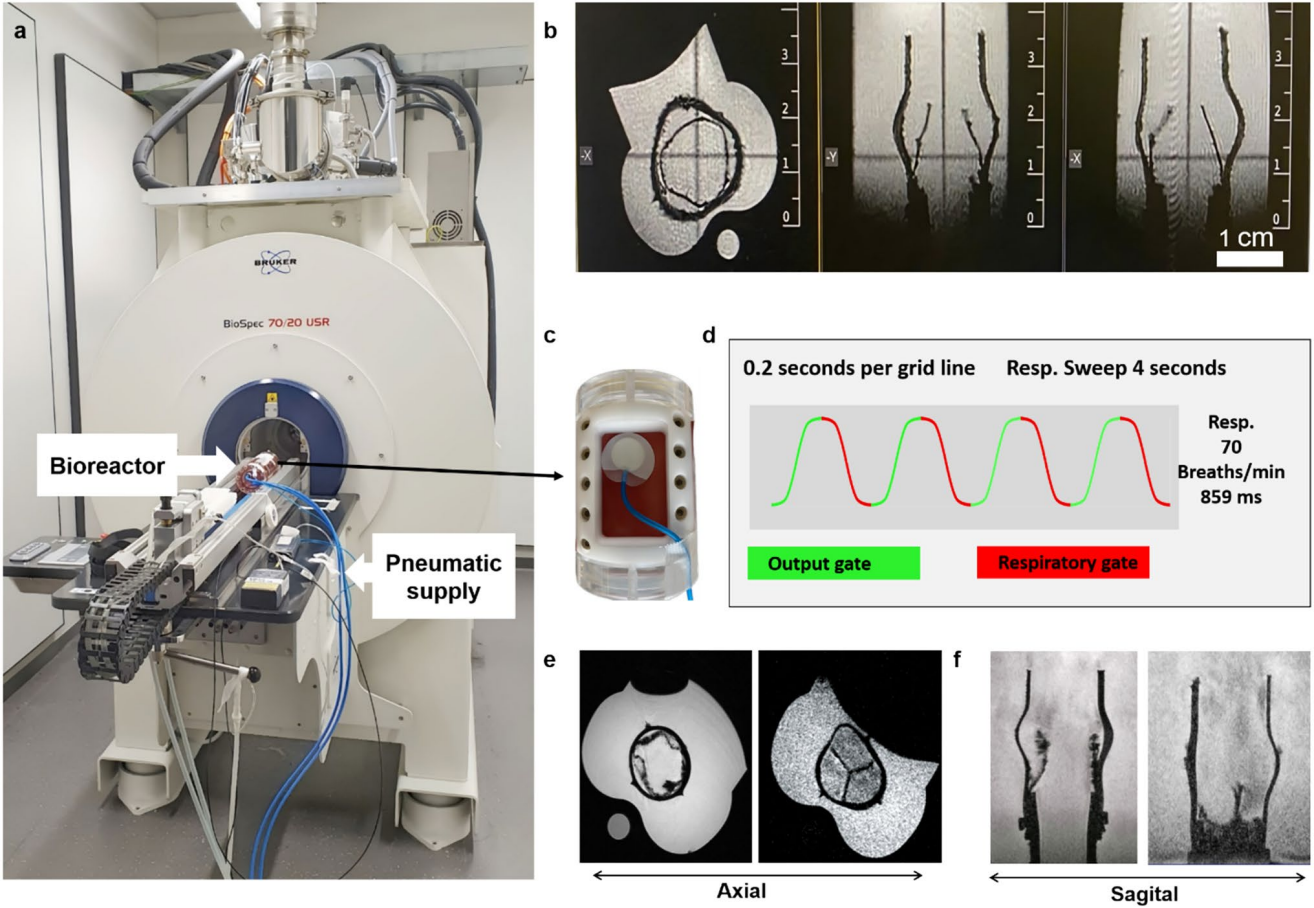
Dynamic magnetic resonance imaging. **a** The bioreactor going inside the 7 T MRI coil and the blue tubes are the inlet and outlet of pneumatic connections. **b** Localizer sequence shows the placement of the valve in MRI. **c** The breathing patch on the top of the ultrasound window. **d** The respiratory graph observation during the MRI scans. **e**–**f** Observation of the opening and closing of the valve in the MRI

Upon placing the bioreactor within the MRI coil (Fig 5a), the initial localizer MRI sequence revealed the placement of the valve and the designated imaging area of interest (Fig 5b). Gated MRI ensured that the images were acquired at consistent and predetermined points in the cardiac or respiratory cycle, allowing for clearer and more accurate visualization of moving heart valves (Fig 5e, f). The breathing patch (Fig 5c) was utilized in this study to detect the cardiac cycles of the TEHV, which were imposed by the pressure supplied through the air chamber. This enabled the performance of gated MRI, which is fundamental for minimizing motion artifacts and obtaining high-quality images. Consequently, the MRI image acquisition was synchronized to the breathing rate, which was set to 70 per minute (Fig 5d).

**Fig. 6:**
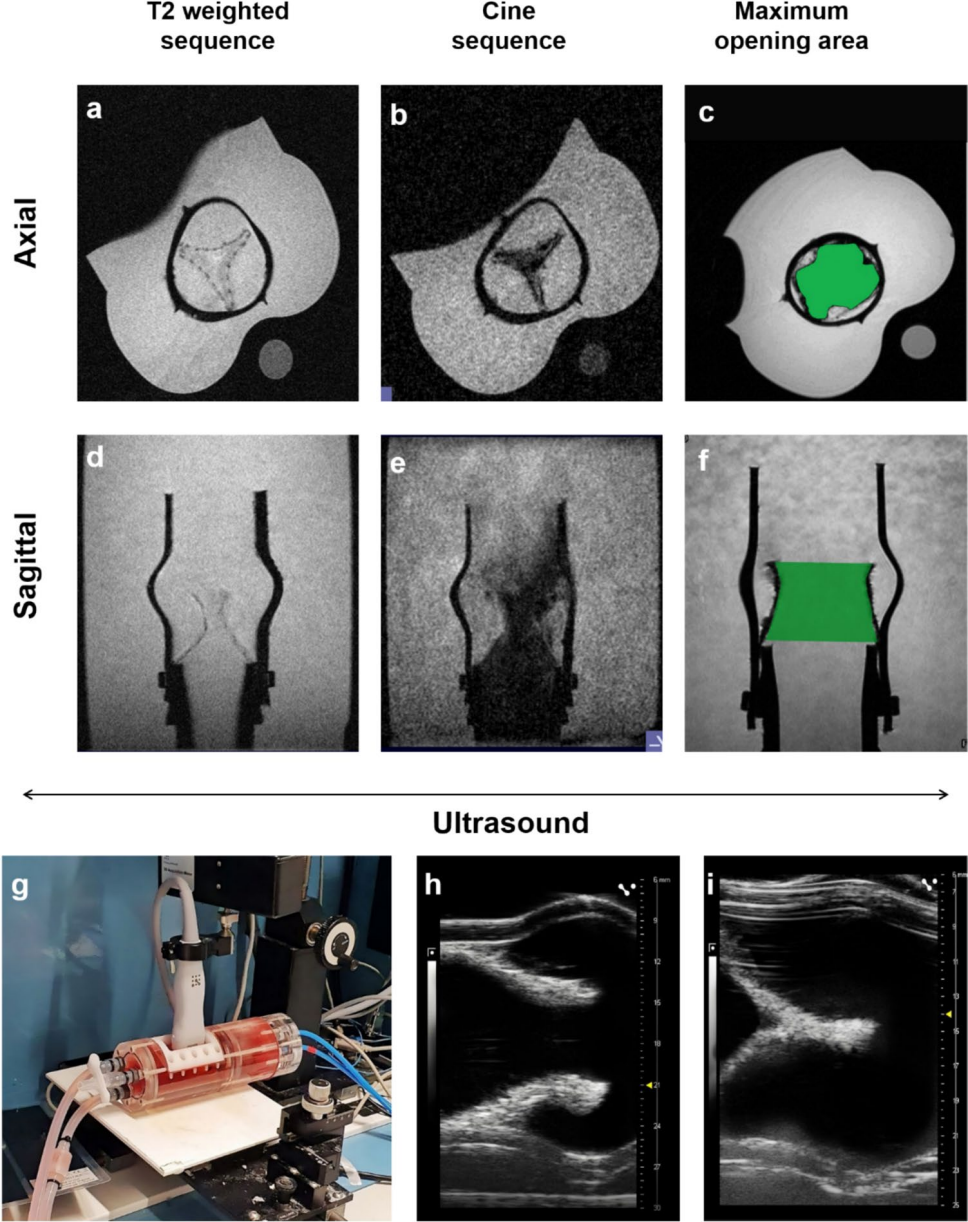
MR and ultrasound imaging of the TEHVs. **a**–**c** Axial views of the TEHVs in both T2-weighted, cine sequence, and maximum opening area. **d**–**f** Sagittal view of the TEHVs in both T2-weighted, cine sequence, and maximum opening area. **g** The bioreactor setup is on the ultrasound bench where the transducer is on a movable axis. **h** Opening frame of the TEHVs. **i** Closing frame of the TEHVs

For qualitative analysis of TEHVs, T2-weighted images were acquired in coronal, sagittal, and axial planes, successfully showing the positioning and dynamic function of the valve (Fig 6a–f). The maximum valve opening area was calculated in comparison to the area of the silicon conduit. The average opening area of the TEHVs (*n* = 3) was determined to be 71% (108.78 mm^2^) when measured from axial MR images (Fig 6c) and 77% (122.82 mm^2^) when measured from sagittal MR images (Fig 6f). These measurements were taken by comparing the opening area to the original diameter area of 15 mm. The standard deviation was 0.85 for the axial measurements and 3.08 for the sagittal measurements. All the samples were tested on day 7 of the dynamic conditioning. These sequences were acquired in both axial and coronal orientations employing a fast spin echo sequence, and this detailed approach provided a comprehensive assessment of the valve’s spatial orientation and visibility within the MRI.

By analyzing the forward and backward volumes during single cycles and calculating the regurgitation factor in accordance with ISO 5840-1, we determined that the regurgitation factor was 12.29% (*n* = 3, SD = 0.85).

Following this initial examination, we further refined our evaluation by employing gradient-recalled echo pulse sequences, commonly known as cine sequences, specifically focusing on the heart valves. These sequences consist of multiple static images that were rapidly displayed in a continuous loop, offering a dynamic portrayal of the cardiac valves. The imaging approach allowed us to capture intricate details of the heart valves in motion, facilitating a comprehensive understanding of their dynamic behavior and structural integrity.

### Ultrasound

The dynamic functionality of TEHVs within the bioreactor was comprehensively tracked using brightfield-mode ultrasound, as depicted in Fig. 6g–i. The transducer was positioned vertically above the ultrasound window, which was a membrane located 2 cm from the heart valves. To reduce acoustic impedance, the small cavity between the membrane and the outer area of the ultrasound window was filled with water. Gray scale values at the opening frames were measured at 144.41 ± 33.42 (a.u), and at closing frames, they were measured at 164.5 ± 37.55 (a.u). This imaging modality facilitated qualitative observation of the opening and closing cycles of the TEHVs, providing valuable insights into their dynamic performance.

### Immunohistochemistry

**Fig. 7:**
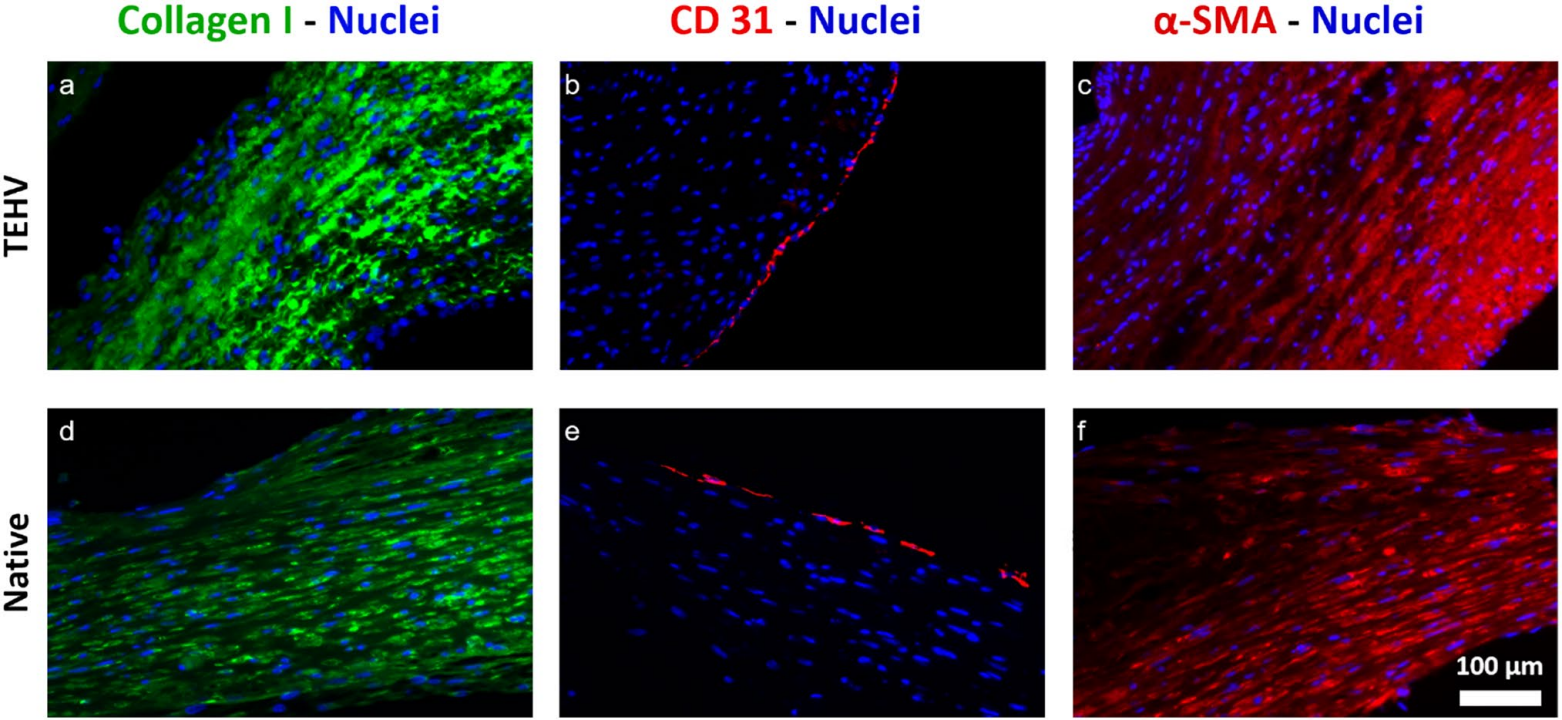
Immunohistolgy analysis of the TEHVs in comparison to porcine aortic valve. **a**–**c** Nuclei (blue), collagen I (green), CD31 (red), α-SMA (red). **d**–**f** Porcine aortic valve as native control

The results of immunohistological staining displayed the expression of collagen I and smooth muscle actin throughout the cusp of the valve, particularly in close proximity to the nuclei (Fig 7a–c). A monolayer of endothelial cells was found on the border of all the TEHV cusps evidenced by CD31 staining (Fig 7b). Furthermore, the TEHVs were qualitatively compared with native porcine aortic heart valves (Fig 7d–f). The TEHVs were not negatively affected by the electrospun coating of SPION-labeled PLGA.

**Fig. 8:**
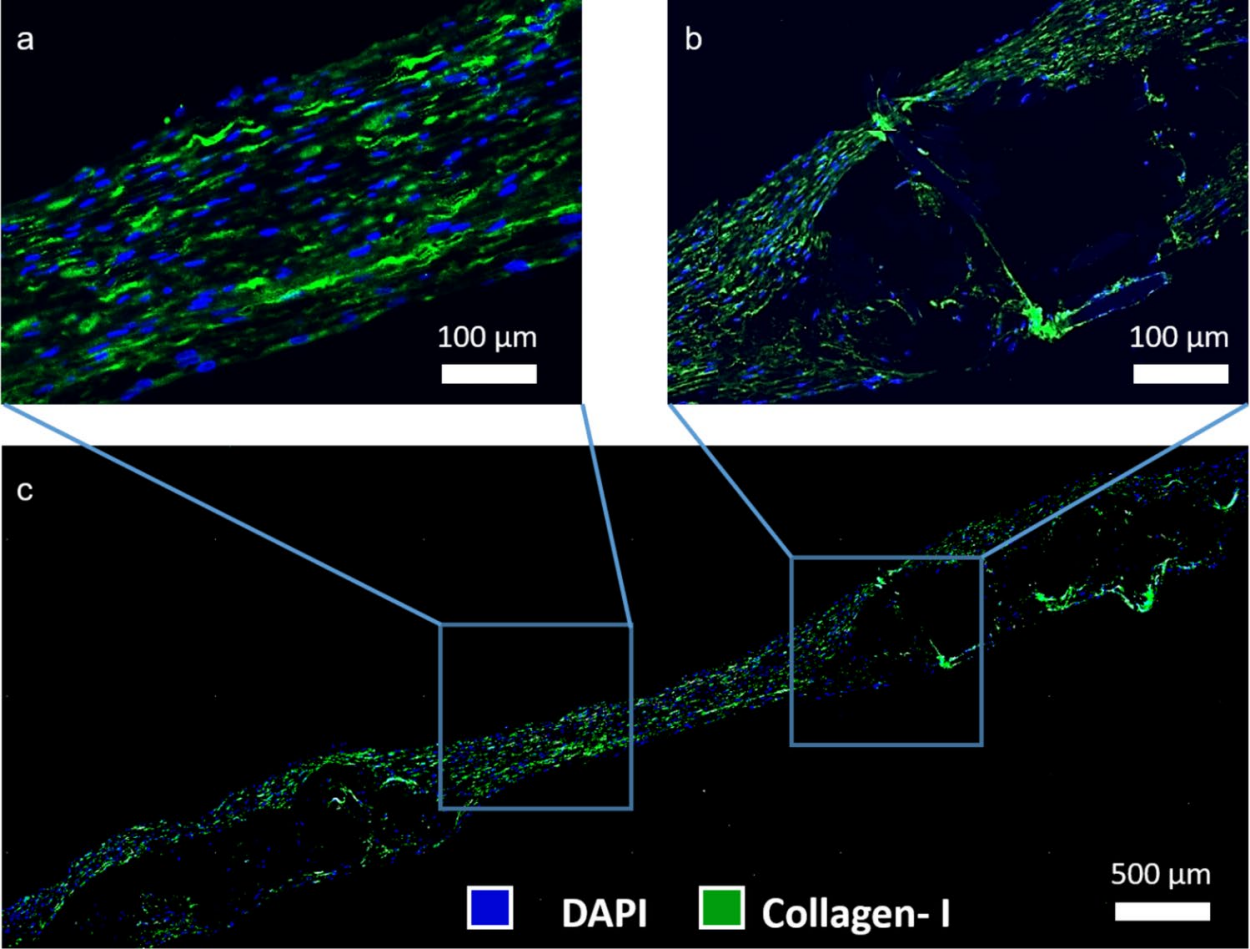
Cell distribution over the cusp. **a** Cell dense areas of the TEHVs. **b** Multifilament textile areas. **c** Overview of the TEHV cusp

An overview observation of the entire cusp of the TEHV shows two different types of cell density areas. The first one, as depicted in Fig 8a, shows the area where the hydrogel was present, and the second type (Fig 8b) shows the area where the multifilament textile was present.

## Discussion

In this study, we designed and constructed a bioreactor with a focus on magnetic resonance imaging (MRI) and ultrasound (US) compatibility. This was achieved by refraining metal components, integrating backflow design, and incorporating a US-compatible membrane, enabling comprehensive imaging of implant behavior without interference. We produced small tissue-engineered heart valves (TEHVs) with a 15 mm diameter, with a specific focus on research for young patients. The TEHVs were produced using polyethylene terephthalate (PET) as the non-degradable backbone of the textile scaffold, poly(lactic-co-glycolic acid) (PLGA) as the biodegradable and biocompatible textile component, and fibrin gel containing smooth muscle cells (SMCs) and endothelial cells (ECs). The TEHVs were dynamically conditioned in a custom-made MRI-compatible bioreactor (Fig. 4, S2) and monitored using MRI and ultrasound imaging, with a focus on assessing implant safety and optimizing life-cycle management.

Several heart valve bioreactors have already been reported in the literature. For example, Amadeo et al. showed a heart valve bioreactor consisting of a decellularized porcine pericardium, in which they were able to achieve successful cell seeding. However, this model was a perfusion-assisted bioreactor, and the achieved flow of 0.03 ml/min is not an optimal human diastolic blood flow rate. The perfusion syringe contained 6 ml of medium, which was changed every 72 h, which is also not an appropriate volume to functionalize a heart valve [34]. A similar attempt was made by Ott et al. with decellularized rat hearts cultured in a perfusion bioreactor system under organ culture conditions [35]. Several other studies reported the use of functional bioreactors to condition heart valves [16, 36]. However, to advance heart valves toward clinical implementation, there is a critical need for robust, longitudinal, non-invasive analysis that can ensure safety and facilitate regulatory approval processes more effectively.

In the present work, we built on our previous efforts where we used the concept of biohybrid tissue-engineered constructs, which consist of fibrin, human cells, and a non-degradable textile scaffold [9, 11]. PLGA fibers were additionally labeled with SPIONs to enhance MRI visibility of the valves and allow for dynamic monitoring of their complex movement. PET was chosen as the scaffold due to its proven biocompatibility, uniformity, mechanical strength, and abrasion resistance [37]. Similarly, PLGA was selected for its proven biodegradability and biocompatibility [38]. Furthermore, the degradation rate of PLGA can be precisely controlled by adjusting the lactic-to-glycolic acid ratio, providing additional flexibility in tailoring the coating’s lifespan to suit specific clinical requirements. This versatility, combined with PLGA's well-established safety profile and mechanical properties, made it the most suitable choice for our coating application, especially when compared to slower-degrading polymers [39, 40].

Upon production, the heart valve undergoes conditioning within a bioreactor (Fig 4d), facilitating optimal flow while regulating pressure and temperature. This bioreactor is meticulously engineered to provide electronic feedback (Fig 4c) through integrated sensors. Moreover, non-invasive imaging techniques are employed to dynamically visualize its function. In our current study, we present the design of a bioreactor with dual objectives: first, to foster tissue maturation in a tissue-engineered heart valve, and second, to enable observation of the valve via MRI and ultrasound modalities.

In the clinic, aortic valve diseases are diagnosed by echocardiography, CT, ultrasound, and MR imaging, which also need to be studied thoroughly in research to observe the remodeling. For tissue conditioning in TEHVs, an adequate environment and specific process parameters are essential. To monitor the conditioning TEHVs, a heart valve bioreactor is equipped with multifunctional setups like motors, pressure sensors, flow sensors, temperature sensors, and many more tubular connections for input and output supply. However, this complicated setup generally contains electronic instruments and metal-containing devices, making it a bottleneck to perform MRI. The characterization of implants through molecular imaging provides the most suitable tool to study implant behavior before they go to transplantation [41]. Backflow is critical in heart valve bioreactors as it affects valve efficiency and durability by simulating physiological regurgitation. We have installed a non-return valve (Fig 4d) to prevent the medium from flowing back into the chamber. By analyzing the flow profile, we calculated the regurgitation factor to be approximately 12.29%. In the future, we plan to apply Doppler flow data to analyze our regurgitation using the ultrasound membrane window in our system.

Bioreactors with mechanical pistons and motors with gear head assembly were also reported before by Miller et al. [42], which could cause the opening and closing of the valve but limited monitoring and molecular imaging ability. Voss et al. and Moreira et al. produced bioreactors with ultrasound windows but involved actuators to move the membrane, for which the MR imaging ability was restricted [24, 29]. We reported a pneumatic system where an elongated tube supplies the air pressure from outside the MRI room (Fig 1e, 5a). With the pneumatic system, the compressor and controller were kept outside the MRI room, and the compressed air reached the bioreactor, which avoided the metal and electronic interference with the MRI. Though the air pressure-actuated heart valve reactor was previously reported by Beelen et al, the bioreactor was very big and complex to get inside a 7 T MRI coil [43]. Furthermore, our integrated backflow design not only makes the bioreactor more compact but also circumvents geometric complexities. Initially, we attempted to fasten the bioreactor using brass and polyamide screws. However, we encountered challenges with brass screws producing artifacts in imaging and polyamide screws proving insufficient due to their low mechanical strength, leading to wear after a single use. This limitation prompted us to transition to PEEK screws, which offered superior mechanical properties and durability for sustained performance within the bioreactor system. The primary novelty of our bioreactor lies in its ability to dynamically image a heart valve under 7 T MRI, a feat that has not been previously reported. The 7 T MRI represents state-of-the-art technology, currently used only in preclinical testing due to its very small-bore diameter.

Our innovation addresses this limitation by designing a compact bioreactor that houses all necessary equipment internally without relying on external tubular connections between the ventricular chamber and aorta. We introduced an integrated backflow technology that allows the medium to circulate internally, enhancing functionality while fitting within the confined space of the 7 T MRI.

As shown in Fig 5b, the valves were initially scanned with the localizer sequence to bring them to the required position where the valve was visible. The static images were taken with the T2-weighted sequence, where the valves were studied qualitatively. Previously, we have proven static non-invasive imaging in tissue-engineered vascular grafts where the structure was enclosed with cylindrical silicon tubes [10, 11].

Compared to vascular grafts, heart valves are more complex and dynamic. Therefore, in the current study, the dynamic movement was studied by executing a cine sequence where the valves could be seen moving, most importantly, the complete opening and closing (Fig 5e, f). Moreover, from a future perspective, MR fingerprinting (MRF) could provide enhanced tissue diagnosis, treatment monitoring, and tissue characterization. MRF is a new approach that allows simultaneous measurement of multiple tissue properties in a time-efficient manner by varying acquisition parameters, such as radiofrequency flip angle, TR, and k-space sampling trajectory [44].

Moroz et al. described how dynamic MRI could answer many clinical questions and allow the study of physiological properties in both normal and diseased tissue constructs [45]. We included a breathing patch (Fig 5c) at the ultrasound window membrane, which showed the breathing rate and allowed motion compensation to avoid motion artifacts. The breathing patch typically consists of a sensor or device placed on the patient’s chest or abdomen to monitor respiratory motion. The information from the breathing patch is then used to trigger the gating of MRI sequences, ensuring that images are acquired at specific points in the breathing cycle. The heart valve’s maximum opening area was observed to range from 70 to 80% compared to its silicone housing (Fig 6c, f), potentially influenced by the valve’s smaller size (15 mm diameter). Effective suturing technique is critical for optimizing valve opening and closure dynamics, suggesting that enhancements in suturing methods could further improve performance. In healthy adults, the effective orifice area (EOA) of the aortic valve typically ranges from approximately 2.5 to 3.5 cm^2^, which is essential for distinguishing normal valve function from pathological conditions [46, 47]. For prosthetic heart valves, the EOA varies based on the type of valve. For example, Firestenberg et al. reported EOAs ranging from 1.3 to 2.1 cm^2^ for bioprosthetic valves [48], while Amarelli et al. found EOAs between 1.5 and 2.0 cm^2^ for mechanical prosthetic valves [49]. Similarly, Buchanan et al. discussed transcatheter aortic valve implantation (TAVI) with EOAs also ranging from 1.5 to 2.0 cm^2^ [50]. To express these values as a percentage of the fully open area of a normal valve, the following formula can be used: Opening Percentage = (Effective Orifice Area/Anatomical Orifice Area) × 100 [51]. For instance, if a patient has an anatomical orifice area of 3.5 cm^2^, an EOA of 2.0 cm^2^ would represent approximately 57% of the fully open area of a normal valve. Given that the anatomical area typically ranges from 2.5 to 3.5 cm^2^, the percentage range for prosthetic EOAs would be around 37% to 84% of the normal valve’s opening area.

The ultrasound results show that the TEHV could be observed and imaged dynamically (Fig 6 g–i). Though ultrasound imaging-assisted bioreactor has been found in the literature, the image quality was found to be distorted due to the thicker membrane and stand-off distance of the transducer from the valve [24, 29]. Hurtado–Aguilar et al. previously constructed a PMMA bioreactor and utilized ultrasound probes to visualize TEHVs. However, the thickness of the reactor wall (ranging from 5 to 15 mm) led to compromised image quality [52]. To address this issue and achieve better visualization of the valves, we designed a dedicated ultrasound window featuring a 0.44 mm-thick membrane secured with PEEK screws (Fig 4d). The use of a 21 Hz transducer combined with a highly elastic membrane enabled easy localization of the valve and yielded high-quality images (Fig 6g–i). Ultrasound analysis, leveraging gray-scale values, offers insights into collagen distribution and formation. Kreitz et al. conducted an analysis correlating gray-scale values from ultrasound scans of cell-embedded fibrin gels with hydroxyproline content, demonstrating a strong association with collagen formation in heart valves, which was consistent with histological findings [53]. This approach can be applied within our bioreactor system and in future longitudinal studies to assess collagen propagation and provide valuable data on tissue development over time. Further, longitudinal studies are needed to assess whether our novel bioreactor model and selected conditioning parameters support in vitro ECM production and deposition, which could be non-invasively monitored by correlating ultrasound gray values with subsequent histological analysis.

The immunohistology analysis meets the hypothesis of having high cell density and high expression of collagen and smooth muscle actin. As depicted in Fig 7, TEHVs exhibit abundant collagen and smooth muscle actin production, with cellular and extracellular matrix (ECM) morphology comparable to that of the native heart valve when observed qualitatively. Further denser ECM can be achieved by prolonged bioreactor conditioning. Nevertheless, our objective is also tied to reducing the conditioning duration to expedite the availability of the valves in clinical settings. The heart valve cusp overview reveals two distinct tissue density regions, illustrated in Fig 8a–c, wherein one area exhibits high density while the other displays a wider blank space. To better understand the phenomena, Fig. 8c and Fig. 3 need to be compared, where the structure of the wrap knitted textile shows the gap between the fibers. The space between the fibers contained most of the hydrogel, which acted as a cell carrier, and the multifilament fibers caused the blank wide spaces in immunohistological analysis.

To summarize, the successful translation of TEHVs into clinical practice necessitates a comprehensive approach to monitoring their integration dynamics and long-term performance. Non-invasive and radiation-free imaging modalities emerge as indispensable tools in this endeavor, offering unparalleled insights into the implant’s life cycle from fabrication through to patient follow-up evaluations. By implementing advanced imaging techniques alongside innovative tissue engineering and bioreactor setup strategies, the implants can pave the way for safer and more efficacious solutions in cardiovascular regenerative medicine. Looking forward, the new bioreactor presents opportunities to explore a wide range of imaging applications for assessing ECM dynamics, scaffold degradation, and relevant markers, providing critical insights into tissue maturation and implant performance.

Our study introduces an innovative bioreactor capable of conditioning a biohybrid heart valve and facilitating multi-modal imaging, including dynamic imaging using a 7 T gated MRI and a 21 Hz ultrasound probe. The adaptation of integrated backflow design and pneumatic control makes the bioreactor physiologically capable of being studied dynamically in a 72 mm bore MRI device. Using a combination of non-degradable scaffolds and cell carrier hydrogels, we have shown a high cell density of the heart valve, confirmed by immunohistology. Consequently, our imaging approach and bioreactor design have the potential to greatly enhance quality control during the crucial transition from advanced in vitro bioreactor maturation to the initial in vivo implantation of cardiovascular implants. Ultimately, this work could contribute significantly to advancing clinical applications and improving patient safety.

## Supplementary Information

Below is the link to the electronic supplementary material.

Supplementary file1 (PDF 362 kb)
