# Supplementary material for: Novel Bioreactor Design for Non-invasive Longitudinal Monitoring of Tissue-Engineered Heart Valves in 7T MRI and Ultrasound
Source: Ann Biomed Eng. 2024 Oct 22;53(2):383–97. doi: 10.1007/s10439-024-03632-8 (PMC11805768; doi:10.1007/s10439-024-03632-8)
Supplement: Supplementary file 1 — Supplementary file1 (PDF 362 kb) [file 10439_2024_3632_MOESM1_ESM.pdf]

Supplimentary data:

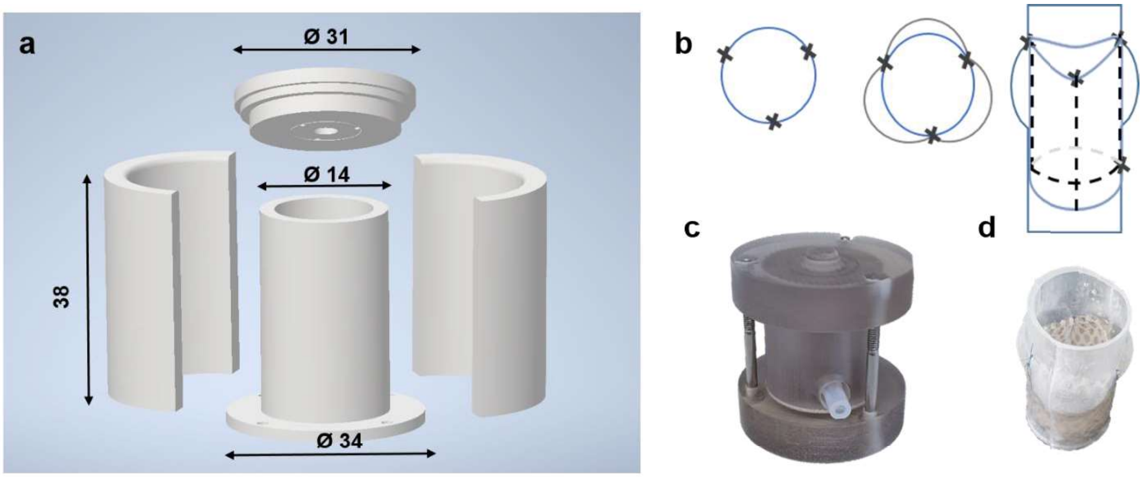

Figure S1: The mold and suturing of the heart valve: a) CAD images of different parts of the mold b) Suture points of the heart valve with the silicon housing c)The mold after 3Dprinted and fastened d)The heart valve inside a silicon housing after suture

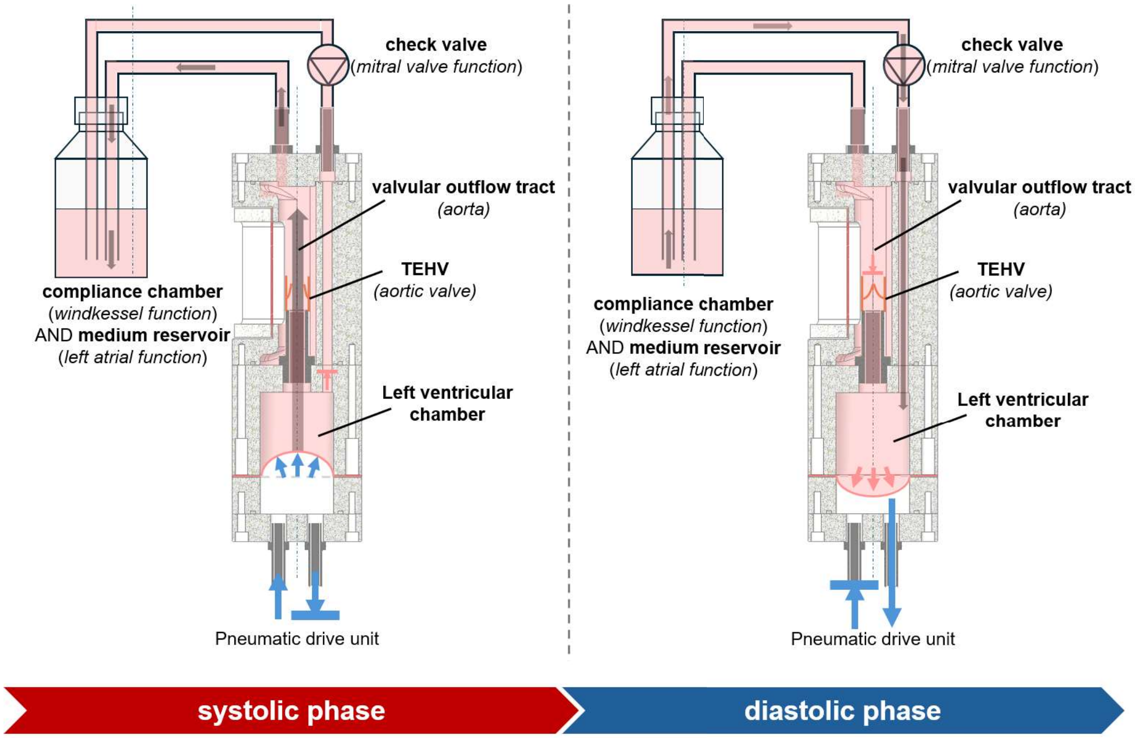

Figure S2: Principles of the bioreactor function in systolic phase and diastolic phase
